# Automatic Approach Tendencies toward High and Low Caloric Food in Restrained Eaters: Influence of Task-Relevance and Mood

**DOI:** 10.3389/fpsyg.2017.00525

**Published:** 2017-04-11

**Authors:** Renate A. M. Neimeijer, Anne Roefs, Brian D. Ostafin, Peter J. de Jong

**Affiliations:** ^1^Department of Clinical Psychology and Experimental Psychopathology, University of GroningenGroningen, Netherlands; ^2^Center for Eating Disorders, Accare Child and Adolescent PsychiatrySmilde, Netherlands; ^3^Department of Clinical Psychological Science, Faculty of Psychology and Neuroscience, Maastricht UniversityMaastricht, Netherlands

**Keywords:** approach tendencies, approach bias, mood, restrained eating, dieting

## Abstract

**Objective:** Although restrained eaters are motivated to control their weight by dieting, they are often unsuccessful in these attempts. Dual process models emphasize the importance of differentiating between controlled and automatic tendencies to approach food. This study investigated the hypothesis that heightened automatic approach tendencies in restrained eaters would be especially prominent in contexts where food is irrelevant for their current tasks. Additionally, we examined the influence of mood on the automatic tendency to approach food as a function of dietary restraint.

**Methods:** An Affective Simon Task-manikin was administered to measure automatic approach tendencies where food is task-irrelevant, and a Stimulus Response Compatibility task (SRC) to measure automatic approach in contexts where food is task-relevant, in 92 female participants varying in dietary restraint. Prior to the task, sad, stressed, neutral, or positive mood was induced. Food intake was measured during a bogus taste task after the computer tasks.

**Results:** Consistent with their diet goals, participants with a strong tendency to restrain their food intake showed a relatively weak approach bias toward food when food was task-relevant (SRC) and this effect was independent of mood. Restrained eaters showed a relatively strong approach bias toward food when food was task-irrelevant in the positive condition and a relatively weak approach in the sad mood.

**Conclusion:** The weak approach bias in contexts where food is task-relevant may help high-restrained eaters to comply with their diet goal. However, the strong approach bias in contexts where food is task-irrelevant and when being in a positive mood may interfere with restrained eaters’ goal of restricting food-intake.

## Introduction

Restrained eaters try to restrict their food intake to control their weight, but typically have difficulties with this and often indulge in the food they want to avoid, eventually leading to weight gain (Herman and Polivy, 1980). This raises the question as to what mechanisms underlie this seemingly inconsistent behavior. Current dual process models (e.g., Hofmann et al., 2009) propose that behavior is determined by two different cognitive systems that may differentially affect people’s eating behaviors. The ‘reflective system’ is assumed to be responsible for voluntary and largely controlled behaviors, and is assumed to be predictive in situations with enough cognitive resources. The ‘impulsive system’ is responsible for impulsive, automatic behaviors in situations with less cognitive capacity. The impulses are thought to emerge from associations that are based on experience (Hofmann et al., 2009). For example, a person may repeatedly experience a stimulus (e.g., chocolate), followed by a behavior (e.g., putting the chocolate in one’s mouth) and then a consequent emotional response to the behavior (e.g., pleasure). As a result, the mere sight of a chocolate may then automatically elicit positive associations and the tendency to approach the stimulus. These associative processes need no attentional resources to function, and are independent of whether a person consciously endorses or rejects the implication of an associative link (Strack and Deutsch, 2004; Gawronski and Bodenhausen, 2006; Hofmann et al., 2009).

The tendency of restrained eaters to overeat might be mediated by positive automatic associations with food. Especially in conditions of impaired self-control, automatic associations may have a stronger influence on behavior than more deliberate processes. The positive association with food might express itself in automatic approach tendencies, the behavioral tendency to approach or avoid food. As a consequence, these processes might drive them to overeat. For instance, negative affect/stress might impair rational processes, and thereby reduce the ability to resist immediate relief in favor of long-term benefits (Baker et al., 2004). This might help explain why restrained eaters, despite their strong wish to control their eating behavior (deliberate process), so often fail.

Indeed, it has been shown that positive associations with high-caloric food may complicate the restriction of food-intake (Stroebe et al., 2008). However, evidence for the idea that especially high-restrained eaters show positive associations, is mixed (see: Roefs et al., 2011). On the one hand, studies using various indirect measurement paradigms provided evidence indicating that restrained eaters show a positive association with high caloric food (Hoefling and Strack, 2008; Houben et al., 2010). In a similar vein, a study on the automatic behavioral tendency to approach or avoid stimuli as a measure for positive associations with food items have shown that restrained eaters have stronger automatic approach tendencies toward food stimuli than unrestrained eaters (Veenstra and de Jong, 2010). On the other hand, one study did not find a relationship between implicit measures of positive attitudes toward high caloric food and restraint status (Roefs et al., 2005). Furthermore, other studies even found opposite results, indicating stronger negative attitudes toward high caloric food in restrained eaters compared to unrestrained eaters (Maison et al., 2001; Papies et al., 2009). Also a study using a computerized reaction time task in which participants had to approach or avoid food with a joystick, found that dieters showed less approach tendencies toward high caloric food words compared to non-dieters (Fishbach and Shah, 2006).

One possible explanation for the inconsistencies in earlier findings resides in methodological differences. Firstly, the studies used different measures to determine restrained eating. Some used the Restraint Scale (RS; Herman and Polivy, 1980) whereas other studies relied on the Dutch Eating Behavior Questionnaire (DEBQ; Van Strien et al., 1986), which might measure slightly different concepts. For instance, weight fluctuation (actual influence of intention on behavior) is measured in the RS, but not in the DEBQ. In addition, some studies measuring positive associations with food used tasks in which food was task-relevant and where one has to approach or avoid a stimulus on the basis of the absence/presence of food-content (e.g., Fishbach and Shah, 2006), whereas others used a task in which food was task-irrelevant and where one has to approach or avoid a stimulus on the basis of a food-irrelevant feature of the picture (e.g., Veenstra and de Jong, 2010). For instance, participants have to approach or avoid the picture on the basis of the perspective of the picture (top view/side view) and the content of the picture (food/non-food) is not relevant for defining a correct response. The Stimulus Response Compatibility task (SRC; De Houwer et al., 2001), is an example of a task-relevant measure, where the content of the picture (i.e., food/non-food) is relevant to the task. That is, the correct response on each trial is defined by whether a stimulus contains food or not (e.g., participants are instructed to approach food and to avoid non-food). Thus, the participant is actively categorizing pictures as either food or non-food stimuli. The Affective Simon Task manikin version (AST; De Houwer et al., 2001) is an example of a task-irrelevant measure, where response requirements depend on stimulus features that are unrelated to the food/non-food content of the pictures, such as the orientation of the stimulus (top versus side view of the object on the picture; e.g., Veenstra and de Jong, 2010). Thus in this case, a participant does not have to categorize a stimulus as food or non-food for giving a correct response (approach or avoid). A participant could, for example, be instructed to approach top view pictures by moving the manikin toward the picture and to avoid side view pictures by moving the manikin away the picture. Though food versus non-food is task-irrelevant in the AST, the absence/presence of food-content systematically varies over trials, and therefore the effect of the task-irrelevant food vs. non-food content of the pictures on reaction times can be analyzed.

Previous studies examining the relevance of automatic approach tendencies in the context of dysregulated eating behavior used the SRC and the AST interchangeably (Fishbach and Shah, 2006; Veenstra and de Jong, 2010). However, both tasks may assess different aspects of participants’ automatic approach tendencies. Because food is relevant to the task in SRC, this task could model situations where the person is deciding what and how much to eat, such as during a common mealtime. Especially when high caloric food items elicit strong automatic approach tendencies, this may have an influence on people’s food selection that is inconsistent with their diet goal. Conversely, because food is irrelevant to the task in AST, (i.e., the response has to be based on another feature than food/non-food) this task could model situations where the person is performing a different task, such as walking to work, but is tempted to eat, for example, by the smell of bread at the bakery. Thus, in both types of situations, automatic approach tendencies toward high caloric food might interfere with one’s goal to restrict food intake. Yet, especially in the latter context, where the non-food relevant task taxes available sources for cognitive control, the impact of automatic approach tendencies might be relatively large and may eventually lead to overeating (high caloric food) in spite of one’s diet goal.

A second explanation for these inconsistent findings in studies on restrained eating and approach tendencies may be that implicit measures of associations do not reflect a fixed phenomenon but instead are highly context dependent and vary as a function of, for instance, mood. If, for example, a person has learned to eat in situations of negative affect or stress, then negative affect might become automatically associated with eating. Accordingly, high stress levels may elicit stronger automatic approach tendencies for food in restrained eaters. Consequently, self-control may break down and it may drive them to overeat, especially in those situations of negative affect/stress. Various studies have indeed demonstrated that restrained eaters increase their food consumption in response to a negative mood (e.g., Vanderlinden et al., 2004; Yeomans and Coughlan, 2009). Moreover, in contrast to unrestrained eaters, restrained eaters have shown an increase in food intake after various stressful events, such as social stressors (e.g., public speaking), ego-threatening stressors (e.g., unsolvable puzzles linked to intelligence) and daily hassles (see for a review Greeno and Wing, 1994).

However, other lines of research suggest that sadness can induce more analytic and deliberative processing (e.g., activates the slow, deliberative system), whereas happy mood is related to more superficial and automatic judgments. For example, there is evidence that a happy mood is associated with increased automatic stereotyping, which is presumed to be based upon implicitly held associations (Schwarz and Bless, 1991). This would suggest that especially a positive mood might automatically activate food-approach associations. In line with this, several studies point to increased food intake during positive emotions for eating- or weight concerned people (e.g., emotional and restrained eaters). A study on emotional eating and mood showed a significant increase in food intake for emotional eaters in the positive compared to the neutral condition (Bongers et al., 2013). Moreover, unsuccessful dieters were found to be vulnerable for increased eating in response to positive emotions (Yeomans and Coughlan, 2009). Accordingly, it has been argued that positive mood might be an underestimated risk factor for dysregulated (over)eating (Bongers et al., 2013).

Furthermore, it is hypothesized that both positive and negative mood might be involved in craving and intake (Baker et al., 1986). Negative-affect craving would be triggered by a negative emotional response or aversive events, whereas the positive-affect craving system would be activated by positive emotional states or cues paired with eating and its pleasurable or positively reinforcing effects. When activated, both the positive and the negative affect system could induce craving experiences, approach behavior, affect, and corresponding physiological reactions. In line with this, a study showed that restrained eaters showed increased food intake both in negative and positive mood states (compared with neutral mood) (Cools et al., 1992). Taken together, there is evidence that both a negative and a positive mood may promote food intake in restrained eaters. So, both in a positive and in a negative mood (compared to a neutral mood) approach tendencies may be enhanced.

In sum, the present study examined: (1) whether restrained eaters show automatic approach tendencies toward food when food is task-relevant and/or when food is task-irrelevant, and (2) whether the effects vary as a function of caloric value, and (3) whether mood has an effect on automatic approach tendencies toward food, subjective craving, and actual food intake, and whether these mood effects are most pronounced for restrained eaters.

## Materials and Methods

### Participants

Considering that weight concerns and dieting behavior are more prevalent among women than men (Schaumberg and Anderson, 2016), only female participants were recruited. Participants were recruited from a participant-pool consisting of undergraduate psychology students and an (on-line) paid participant-pool administered by the University of Groningen. We included 92 female participants (*M =* 21.5 years, *SD* = 2.14) who received either course credit or money (11 Euro for 1.5 h) as a compensation for participating in our study. Prior to testing, participants were screened for depressive symptoms with the Major Depression Inventory (MDI, Bech et al., 2001). Five of the 92 participants scored above the diagnostic threshold of meeting at least five of the DSM-IV (American Psychiatric Association [APA], 2000) criteria (including sad mood or loss of interest). They completed the study, but were assigned to a positive or neutral mood induction (because a negative mood induction could pose a mental health risk to depressed individuals), and were later removed from the data because randomization was violated. See **Table 1** for a description of the participants.

**Table 1:** Group characteristics of the different mood groups.

	Positive (*n =* 22)	Neutral (*n* = 23)	Stressed (*n* = 21)	Sad (*n* = 21)	Between groups tests	
					*F*	*p*
Age	21.81 (2.51)	21.41 (1.97)	20.88 (1.69)	22.00 (2.42)	0.82	0.49
BMI	21.31 (2.13)	21.96 (3.88)	23.32 (4.28)	22.21 (2.64)	0.84	0.48
RS score	12.82 (4.86)	13.13 (4.96)	14.81 (6.05)	13.81 (6.09)	0.55	0.65
Liking HC	38.81 (7.93)	38.74 (9.24)	40.24 (7.32)	37.90 (7.97)	0.30	0.83
Liking LC	31.72 (5.04)	31.30 (6.03)	31.19 (5.01)	28.81 (8.88)	0.90	0.44
Wanting HC	29.04 (9.40)	23.82 (11.78)	25.52 (10.75)	28.10 (8.29)	1.22	0.31
Wanting LC	28.14 (7.74)	25.35 (7.59)	24.62 (4.95)	23.52 (8.87)	0.91	0.44
Frequency HC	27.09 (6.05)	28.13 (7.21)	26.52 (6.41)	24.76 (8.53)	0.86	0.47
Frequency LC	27.50 (5.89)	27.52 (6.45)	27.05 (6.59)	25.29 (7.66)	0.54	0.66
Stressed T1	1.97 (0.84)	1.83 (0.67)	2.04 (0.70)	2.17 (0.64)	0.49	0.69
Sadness T1	1.64 (0.99)	1.37 (0.79)	1.54 (0.64)	1.36 (0.46)	0.37	0.78
Happiness T1	2.82 (0.64)	2.87 (1.05)	2.46 (0.59)	2.64 (0.82)	0.41	0.75
Total amount eaten	39.09 (26.73	30.05 (29.16)	32.19 (18.20)	32.43 (18.22	0.60	0.62

*Mean characteristics, with *SD* in parentheses; HC, high caloric; LC, low caloric; BMI, body mass index; RS, restraint scale. T1 is prior to the mood induction.*

### Stimulus Selection

Stimuli for the AST and the SRC tasks were adapted from Veenstra and de Jong (2010), with some modifications. The stimuli consisted of eight high-caloric food pictures (pizza, croissant, chocolate, chips, fries, ice-cream, cookie, and toast with ham and cheese), eight low-caloric food pictures (strawberries, melon, carrots, cherries, cucumber, tomato, apple, bell pepper), and eight neutral stimuli (various office items). For the AST and the SRC two different stimuli sets were used. For the AST, two different pictures (380 × 285 pixels) were constructed for each type of these food items: one displaying the food from a top view and one from a side view. Pictures were shown in a way it was clear whether it was top view or side view (e.g., showing it on a plate).

### Materials

#### Computer Tasks

To test if restrained eaters’ automatic approach tendencies are most pronounced when food is task-irrelevant (AST; cf. Veenstra and de Jong, 2010), or whether approach tendencies might also be non-intentionally activated in conditions when food is task-relevant, the current study included both types of tasks. Both high and low caloric food pictures were included to explore whether the effect would be most pronounced with high caloric food pictures. The computer tasks were programmed in E-prime 2.0 (Schneider et al., 2002) and administered on a Windows XP computer with the screen resolution set to 1280 by 1024 pixels.

#### AST

As an index of automatic approach tendencies we used a manikin task that was based on the AST originally developed by De Houwer et al. (2001). The AST was adapted from Veenstra and de Jong (2011) with minor modifications, including switching the language of instructions from Dutch to English. Each trial started with a 1000-ms presentation of a fixation dot. Next, a picture appeared in the middle of the screen, and a black manikin appeared above or below the picture. Participants in the AST-manikin had to move the manikin toward or away from the picture by (repeatedly) pressing the arrow buttons. The picture remained on the screen until the manikin had reached the picture or the edge of the screen. The required response (move toward or away) was defined by the perspective of the picture (top-view versus side-view). The content of the stimuli (high-caloric food, low-caloric food, or neutral pictures) was a task-irrelevant stimulus feature and could thus be ignored by the participant for correct task-performance.

The AST consisted of a practice block of eight trials, followed by two test blocks of 96 trials each. Trials differed in stimulus type (i.e., task-irrelevant feature: high caloric, low caloric, and neutral), the side from which the photograph was taken (i.e., task-relevant feature: top-view vs. side view), and position of the manikin (i.e., above or below the picture). Each stimulus was presented four times in each block (top view: manikin above; top view: manikin below; side view: manikin above; side view: manikin below). For each participant, trials were presented in a unique random order. Half of the participants were instructed to move the manikin toward top views and away from side views, and half of the participants were instructed to move the manikin toward side views and away from top views. Furthermore, participants were instructed to move the manikin as fast and accurately as possible.

#### SRC

The SRC task was programmed and executed in a similar way as the AST. The central difference was that in the SRC, participants had to approach or avoid according to the content of the picture (i.e., food vs. non-food). The participants were instructed to approach food (high and low caloric) and avoid non-food items in one block, whereas the response assignment was reversed in the other block (avoid food and approach non-food items). The order of the blocks was balanced over participants: Half of the participants had to approach food in the first block, and avoid in the second. For the other half of the participants, the instructions were reversed (first avoid food, then approach it). The food SRC consisted of two practice blocks with four trials each and two test blocks of 96 trials each.

#### Mood Induction

To control for mood and test whether mood would influence automatic approach tendencies and eating, a mood induction was carried out. Earlier research often used a sad mood induction, but other dimensions of a negative mood, as for instance stressed mood, may also be involved. Therefore, four mood conditions were used (sad, stressed/anxious, positive, and neutral). The mood induction included imagery and music components. The imagery component of the mood induction was adapted from Sinha et al. (2009). Participants were instructed to identify and write about a situation and about what they had experienced in the described event. Participants in the neutral condition had to write about a typical day. They were asked to describe both the environmental details of the event/day and emotions that they experienced during the event. After writing about these details, participants were presented with a list of bodily sensations (e.g., heart pounds, breathes faster) and were instructed to indicate which of these they had experienced in the event they just described. Participants imagined the event for 5 min with instructions to imagine the event as if it was happening in the present. While imagining the event, participants also listened to music corresponding to the mood condition. The music used in this experiment has been shown to be effective for mood inductions in previous studies (e.g., Bradley et al., 2007). Participants in the positive mood condition listened to Grieg’s ‘Morning Mood’ from ‘Peer Gynt’ while those in the neutral condition listened to ‘Neptune’ from Holst’s ‘The Planets.’ In the sad mood induction, participants listened to ‘Russia under the Mongolian Yoke’ by Prokofiev at half speed, and participants in the stressed/anxious mood condition listened to ‘The Rite of Spring’ by Stravinsky.

#### Taste Test

Food intake was examined with a bogus ‘taste test.’ In this task participants were instructed to taste several kinds of high caloric snack foods and judge the food on various aspects such as quality and saltiness. Unbeknownst to the participants, total food intake was measured (in grams). The food consisted of four high-caloric snacks: chocolate nuts (508 Kcal/100 g), potato chips (545 Kcal/100 g), rice crackers with chocolate (376 Kcal/100 g), and salty sticks (490 Kcal/100 g). The snacks were presented in white plastic food containers that were placed in a box on the participant’s table. The box was closed so that participants were unable to see the snacks before the taste test began. Participants were told that the researchers were interested in their taste perception at that moment to reduce suspicion that their food intake would be calculated afterward. They were given 7 min to complete questions about the food’s taste and quality (e.g., “How would you judge the quality of the potato chips?”; “To what extent do you think the potato chips are salty?”). Participants were told they could taste as much as they wanted during the task. Participants were allowed to eat as well during the subsequent tasks until the end of the experiment. Throughout the taste task, participants listened to the music linked to their mood condition (as described above). The food was weighted before and after the experiment to calculate the total amount of food that was consumed.

### Questionnaires

#### Restraint Scale

Dietary restraint was assessed by the RS (Herman and Polivy, 1980). The scale consists of 10 items (e.g., “How often are you dieting?”) and its scores range from 0 to 35, with five questions ranging from one to four, and five questions ranging from one to three. Higher scores refer to attempts to control weight.

#### Visual Analog Scales (VAS)

As an subjective measure of approach tendencies, participants’ craving for food stimuli was assessed. Furthermore, participants’ liking, as well as participants’ frequency with which they ate the food items represented in the pictures that were used in the implicit measurement procedures, were assessed. To index craving we asked: ‘How much do you crave this product at this moment?’ Liking of food items of the AST was assessed by answering the question: ‘How much do you like this product?’ To assess the frequency with which they ate the particular food we asked ‘How frequently do you eat this product.’ The questions were answered on a VAS ranging from 0 (not at all) to 100 (very much/very often).

#### Mood Questionnaire

To measure the current mood state, participants indicated on a scale from one (very slightly or not at all) to five (extremely) how much they felt that way right now, that is at the present moment, in response to 15 mood-describing adjectives. Three adjectives were taken to describe each (except neutral) mood condition (positive: happy, glad, elated; sad: sad, down, depressed; stressed: stressed, on the edge, nervous), and six unrelated mood adjectives (e.g., proud) derived from the PANAS scale (Watson et al., 1988) were added.

### Procedure

**FIGURE 1:**
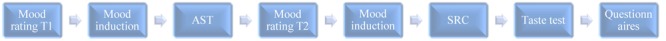
**An overview of the experiment**.

See **Figure 1** for an overview of the experiment. Participants were asked to abstain from eating for 1 h before the experiment. To control for a decreased desire to eat sweets early in the morning as compared to the rest of the day, the study took place in the late morning and afternoon. Participants were tested in groups of a maximum of six participants. On arrival, they completed the informed consent form. The participants were randomly assigned to the positive, neutral, sad, or stressed/ anxious mood condition. Participants were seated next to each other, but were separated by partition walls so they could not see each other. They were asked to wear headphones throughout the experiment to ensure that they could not hear each other and would be focused on the tasks.

At the beginning of the experiment, participants completed the mood questionnaire to assess their current mood state. Then, participants followed the mood induction as described above. After this, participants performed the AST. The AST was administered first (before the SRC), because we anticipated less carry-over effects from a task with food as task-irrelevant feature to a task with food as a task-relevant feature than vice versa (e.g., Bosson et al., 2000). Next, participants completed the mood questionnaire for a second time to verify the effectiveness of the mood manipulation. This was done after the AST to minimize the time between the induction and the computer task, and thus to optimize the influence of the mood manipulation on task performance. As a booster of the condition-relevant mood, participants were then asked to repeat parts of the first mood induction. This time, the mood induction took 3 min and involved re-living the situation and listening to the music from the first mood induction. Then, participants completed the SRC. Subsequently, participants performed the taste task to assess their level of food consumption. They were given 7 min to complete the food ratings. The condition-specific music continued during the taste task. Next, participants completed the RS. Then, they were asked how much they liked, wanted, and how often they consumed each of the food items that were represented in the pictorial AST and SRC tasks. Finally, participants’ weight and height were measured, to calculate their Body Mass Index.

### Data Reduction

Reaction times (time until first response) were used in the analyses. Trials of the AST and the SRC task with reaction times below 200 ms and trials with errors (trials in which the first response was in the wrong direction; 21 and 12% for, respectively, the AST and SRC) were excluded from the analysis in order to have meaningful trials (following Veenstra and de Jong, 2010). To limit the influence of outliers, the median reaction times per condition per participant were used in the data analysis.

Approach biases were calculated by subtracting reaction times of approach trials from reaction times of avoidance trials for each stimulus type separately (high caloric food, low caloric food, neutral pictures). To calculate the approach biases for high and low caloric food, we subtracted the approach bias of neutral pictures from the approach bias of high caloric and low caloric food pictures, respectively. A positive approach bias indicates a tendency to approach food pictures and a negative approach bias (avoidance bias) indicates a tendency to avoid food pictures.

## Results

### Group Characteristics

Participants in the four mood conditions did not differ in age, BMI, RS, liking for high or low caloric food, wanting for or frequency of eating high and low caloric food, happiness, sadness, and stressed mood, see **Table 1**.

### Manipulation Check

See **Table 2** for mean mood scores. To assess differences in mood between the four mood conditions after the manipulation (T2), data were analyzed in a 3 (mood T2 rating: happiness, sadness, and stressed mood) × 4 (condition: positive, stressed, sad, neutral) mixed ANOVA. There was an interaction effect of mood rating × condition, *F*(6,162) = 2.73, *p =* 0.02, $\eta_{p}^{2}$ = 0.09. *Post hoc* tests indicated that participants in the sad mood condition generally reported higher sadness than participants in the positive condition, *t*(40) = 3.07, *p* < 0.01, *d* = 0.97, whereas participants in the positive condition reported higher happiness scores than in the sad, *t*(40) = 2.15, *p* = 0.04, *d* = 0.68, and the stressed condition *t*(40) = 2.06, *p* = 0.04. *d* = 0.65.

**Table 2:** Means of stressed, sad, and happy mood ratings for each mood condition.

	Mood condition			
	Stressed	Neutral	Happy	Sad
**Mood rating**
Stressed	1.84 (0.87)ˆa	1.68 (0.77)ˆa	1.54 (0.52)ˆa	1.83 (0.92)ˆa
Sadness	1.44 (0.54)ˆab	1.55 (0.86)ˆab	1.21 (0.36)ˆa	1.87 (0.93)ˆb
Happiness	2.19 (0.80)ˆa	2.38 (0.79)ˆab	2.65 (0.64)ˆb	2.21 (0.70)ˆa

*Mean scores with standard deviations in parenthesis for T2 rating (after mood induction and AST). Means are based on a five-point rating scale (ranging from 1 = very slightly or not at all to 5 = extremely). Mean values with the same superscript letters in each row were similar and no statistically significant differences were observed.*

### AST Approach Bias for High Caloric and Low Caloric Food

Means of the median reaction times for each mood condition are presented in **Table 3** and errors percentages in **Table 4**. AST approach bias scores of the reaction times were subjected to a 2 (food type: HC, LC) × 4 (mood condition: positive, sad, stressed, neutral) mixed ANCOVA with centered RS as a covariate. Overall, participants showed an approach bias toward high caloric food, compared to neutral pictures, as was evidenced by a significant intercept, *F*(1,74) = 3.81, *p* = 0.05, $\eta_{p}^{2}$ = 0.08. There was no main effect of mood, *F*(3,74) = 0.55, *p* = 0.65, $\eta_{p}^{2}$ = 0.02, food type, *F*(1,74) = 0.004, *p* = 0.95, $\eta_{p}^{2}$ = < 0.01, or of restraint, *F*(1,74) = 0.003, *p* = 0.96, < 0.01. However, there was a significant mood × restraint interaction *F*(3,74) = 3.64, *p* = 0.02, $\eta_{p}^{2}$ = 0.13, that was independent of food type, as was evidenced by the absence of a mood × restraint × food type interaction, *F*(3,74) = 1.19, *p* = 0.32, $\eta_{p}^{2}$ = 0.05.

**Table 3:** Mean AST reaction times and bias scores as a function of mood.

	Stressed	Neutral	Positive	Sad
High caloric food				
Approach	787 (160)	799 (169)	759 (147)	793 (122)
Avoid	911 (186)	904 (155)	869 (126)	908 (137)
Low caloric food				
Approach	766 (186)	760 (164)	755 (125)	782 (151)
Avoid	901 (181)	864 (143)	876 (131)	873 (122)
Neutral stimuli				
Approach	814 (166)	820 (158)	817 (167)	863 (137)
Avoid	938 (182)	901 (172)	889 (165)	903 (138)
AST Bias scores HC	1 (170)	24 (151)	38 (189)	76 (116)
AST Bias scores LC	10 (187)	23 (174)	49 (125)	53 (202)

*Means of the AST median reaction times and bias scores in ms, with SD in parenthesis. Approach biases were calculated by first subtracting reaction times of approach trials from reaction times of avoidance trials for each stimulus type separately (high caloric food, low caloric food, neutral pictures) and subsequently, subtracting the approach bias of neutral pictures from the approach bias of both high caloric and low caloric food. AST, Affective Simon Task, manikin version; HC, high caloric; LC, low caloric.*

**Table 4:** Mean AST error percentages as a function of mood.

	Stressed	Neutral	Positive	Sad
High caloric food				
Approach	13.52 (8.21)	14.19 (7.92)	13.86 (8.07)	14.16 (5.35)
Avoid	28.33 (14.21)	23.81 (9.85)	28.28 (11.69)	23.11 (14.13)
Low caloric food				
Approach	14.95 (8.33)	13.95 (9.45)	12.19 (9.55)	12.68 (9.74)
Avoid	34.38 (14.68)	30.19 (13.67)	28.57 (14.26)	27.58 (14.39)
Neutral stimuli				
Approach	16.62 (7.61)	19.14 (10.99)	17.47 (8.15)	15.16 (10.46)
Avoid	29.48 (13.07)	27.48 (13.39)	23.71 (8.17)	23.42 (14.99)
AST Bias scores HC	3.10 (10.88)	4.95 (9.94)	3.62 (8.99)	1.00 (7.64)
AST Bias scores LC	1.67 (10.75)	5.19 (11.58)	5.29 (11.14)	2.47 (8.44)

*Means of the AST errors and bias scores, with SD in parenthesis. Approach biases were calculated by first subtracting amount of errors of approach trials from amount of errors of avoidance trials for each stimulus type separately (high caloric food, low caloric food, neutral pictures) and subsequently, subtracting the approach bias of neutral pictures from the approach bias of both high caloric and low caloric food. AST, Affective Simon Task manikin version; HC, high caloric; LC, low caloric.*

To examine the source of the mood × restraint interaction, the correlations between AST approach bias scores and RS-scores were compared across the various mood-conditions with a *t*-test after a Fisher r-to-z transformation. The correlation between AST (HC and LC combined) and level of dietary restraint in the positive mood (*r* = 0.40) differed from the correlation in the neutral (*r* = -0.37), *z* = 2.43, *p* < 0.01 and sad mood (*r* = -0.36), *z* = 2.31, *p* = 0.02. There were no significant differences between the other mood conditions, all *z* < 1.64, all *p* > 0.10. This indicates high that restrained eaters showed relatively strong approach tendencies for food in a positive mood, whereas low restrained eaters showed relatively strong approach tendencies in a neutral and sad mood.

### SRC Approach Bias for High and Low Caloric Food

Means of the median reaction times for each mood condition are presented in **Table 5** and error percentages in **Table 6**. SRC approach bias scores of the reaction times were subjected to a 2 (food type: HC, LC) × 4 (mood condition: positive, sad, stressed, neutral) mixed ANCOVA with centered RS as a covariate. Overall, participants showed an approach bias toward food, compared to neutral pictures as was evidenced by a significant intercept, *F*(1,75) = 26.15, *p* < 0.001, $\eta_{p}^{2}$ = 0.26. There was no main effect of mood, *F*(3,75) = 1.05, *p* = 0.38, or food type, *F*(1,75) = 0.15, *p* = 0.70, $\eta_{p}^{2}$ > 0.01 but the effect of restraint was significant, *F*(1,75) = 4.59, *p* = 0.04, $\eta_{p}^{2}$ = 0.06, indicating that high restrained eaters generally showed less approach tendencies (or more avoidance) than low restrained eaters. There was no significant mood × restraint interaction effect *F*(3,75) = 1.22, *p* = 0.31, $\eta_{p}^{2}$ = 0.05, but there was a trend of food type × mood × restraint interaction, *F*(3,75) = 2.43, *p* = 0.07, $\eta_{p}^{2}$ = 0.09, suggesting that the mood × restrained interaction may differ for both food types. Two ANCOVA’s (for both high and low caloric food) with mood condition as a between group variable, centered RS as a covariate and SRC approach bias as dependent variable were conducted to examine the source of this possible interaction. Only for high caloric food the RS × mood interaction was significant, *F*(4,78) = 2.77, *p* = 0.03, $\eta_{p}^{2}$ = 0.12. Low restrained participants showed relatively weak approach tendencies for high caloric food in the positive mood and relatively strong approach tendencies in the stressed mood while an opposite pattern seemed present in high restrained participants (i.e., relatively weak approach during stressed mood and relatively strong approach tendencies during positive mood).

**Table 5:** Mean SRC reaction times as a function of mood.

	Stressed	Neutral	Positive	Sad
High caloric food				
Approach	568 (79)	574 (140)	551 (73)	597 (108)
Avoid	629 (90)	640 (93)	661 (92)	652 (105)
Low caloric food				
Approach	559 (85)	546 (75)	560 (85)	578 (114)
Avoid	622 (71)	613 (96)	662 (71)	651 (93)
Neutral stimuli				
Approach	652 (80)	625 (86)	652 (80)	670 (109)
Avoid	671 (71)	655 (83	671 (71)	641 (75)
SRC Bias scores HC	43 (155)	36 (217)	110 (92)	83 (125)
SRC Bias scores LC	44 (130)	36 (127)	85 (96)	101 (130)

*Means of the SRC median reaction times and bias scores in ms, with SD in parenthesis. Approach biases were calculated by first subtracting reaction times of approach trials from reaction times of avoidance trials for each stimulus type separately (high caloric food, low caloric food, neutral pictures) and subsequently, subtracting the approach bias of neutral pictures from the approach bias of both high caloric and low caloric food. SRC, Stimulus response compatibility task; HC, high caloric; LC, low caloric.*

**Table 6:** Mean SRC error percentages as a function of mood.

	Stressed	Neutral	Positive	Sad
High caloric food				
Approach	7.15 (6.64)	8.75 (6.41)	9.00 (8.68)	8.90 (8.69)
Avoid	17.35 (13.85)	12.70 (10.26)	13.59 (11.73)	14.10 (9.69)
Low caloric food				
Approach	5.70 (8.57)	5.25 (6.37)	7.68 (6.80)	5.05 (6.39)
Avoid	16.80 (12.26)	17.50 (10.32)	14.91 (10.50)	11.19 (9.41)
Neutral stimuli				
Approach	17.35 (10.99)	18.20 (12.68)	15.41 (10.00)	17.86 (14.26)
Avoid	15.40 (13.62)	13.65 (13.51)	12.09 (6.96)	16.76 (15.38)
SRC Bias scores HC	10.20 (11.81)	9.68 (13.33)	6.43 (12.12)	9.58 (16.37)
SRC Bias scores LC	11.65 (13.47)	13.42 (16.22)	8.48 (8.30)	13.32 (16.53)

*Means of the SRC errors bias scores, with SD in parenthesis. Approach biases were calculated by first subtracting amount of errors of approach trials from amount of errors of avoidance trials for each stimulus type separately (high caloric food, low caloric food, neutral pictures) and subsequently, subtracting the approach bias of neutral pictures from the approach bias of both high caloric and low caloric food. SRC, Stimulus response compatibility task; HC, high caloric; LC, low caloric.*

### Craving and Food Intake

See **Table 1** for mean food intake scores for the different mood groups and craving (wanting) scores. Craving scores and food intake during the taste test were subjected to three ANCOVA’s with condition (positive, sad, stressed, neutral) as a between-subjects factor, RS as a covariate, and craving (high and low caloric food) and food intake as dependent variables. There were no main effects of mood or restraint on participants’ craving of high and low caloric food or total food intake, nor interaction effects of mood × restraint, all *F*’s(3,78) < 2.54, all *p*’s > 0.12.

### Relationships between Approach Tendencies, Craving, and Food Intake

To explore the overall relationships between approach tendencies, subjective craving, and actual food intake we computed (two-tailed) correlations (**Table 7**). There was a positive relationship between AST approach bias for high caloric food and craving for high caloric food. Also for the SRC there was as a positive relationship between approach bias for high caloric food and craving.

**Table 7:** Correlations between approach bias, craving, restraint scale, BMI and amount eaten.

	AST HC	AST LC	SRC HC	SRC LC	Craving HC	Craving LC	RS	BMI	Total amount eaten
AST HC		0.75ˆ**	0.06	-0.04	0.26ˆ*	-0.14	-0.02	-0.21	0.20
AST LC			0.14	0.05	0.28ˆ*	0.21	0.02	-0.12	0.13
SRC HC				0.77ˆ**	0.35ˆ**	0.19	-0.27ˆ*	-0.27ˆ*	0.16
SRC LC					0.33ˆ**	0.05	-0.21	-0.28ˆ*	0.09
Craving HC						0.06	-0.17	-0.38ˆ**	0.09
Craving LC							0.01	-0.04	0.07
RS								0.36ˆ**	-0.15
BMI									-0.21ˆ*

*Positive bias scores indicate stronger approach tendencies toward food. AST, Affective Simon Task-manikin version; SRC, Stimulus Response Compatibility task; HC, high caloric; LC, low caloric; RS, restraint scale; BMI, body mass index. ^∗^*p* < 0.05; ^∗∗^*p* < 0.01.*

There was a trend toward a significant relationship between AST approach bias for high caloric food and total amount eaten during the taste task, *r*(81) = 0.20, *p* = 0.07. There was no significant relationship between AST approach bias for low caloric food and food intake, neither for the SRC high and low caloric food. Finally, attesting to the relevance of differentiating between approach-avoidance tasks in which food is task-relevant versus tasks in which food is task-irrelevant, the SRC and AST indices showed only a very weak association that was not statistically significant.

## Discussion

The current study examined to what extent automatic approach tendencies toward food are influenced by both positive and negative mood, and whether the impact of these mood states would be especially pronounced in participants with relatively high dietary restraint, and especially evident for high caloric food, thereby interfering with the diet-goal of restrained eaters. The major findings were (i) overall, whether the task was food-relevant or not, participants showed a tendency to approach food rather than avoid it; (ii) when food was task-relevant, restrained eaters demonstrated less approach toward food; (iii) in the positive mood condition and when food was task-irrelevant, restrained eating was associated with stronger approach tendencies toward high caloric food; (iv) there was a positive relationship between approach bias for high caloric food and subjective craving both when food was task-relevant and task-irrelevant; (v) there was a trend positive relationship between actual food intake during the taste task and approach bias for high caloric food, when food was task irrelevant.

### Restrained Eating and Approach Bias

Approach bias measured with the AST was not unconditionally related to restrained eating. The finding that the AST approach bias for high and low caloric food was not generally heightened in participants with relatively high scores on the RS seems inconsistent with the previous finding that especially restrained eaters showed a heightened approach bias for food as indexed by the AST (Veenstra and de Jong, 2010). However, there were several differences between the study of Veenstra and de Jong (2010) and the current study, which might help explain the differences in outcomes. Most important, in the current study, participants underwent a mood induction prior to the AST, which influenced the approach bias (see below).

In the SRC, there was a negative relationship between restrained eating and approach bias: low restrained eaters generally showed a stronger approach bias (or less avoidance) than high restrained eaters. This is in line with previous research showing that restrained eaters displayed less approach bias when automatic approach tendencies were measured with a task in which food was a task relevant feature (Fishbach and Shah, 2006). This indicates that the SRC-task more closely reflects behavior consistent with their dieting goal.

### Mood and Approach Bias

Positive and negative mood induction differentially affected the relationship between dietary restraint and the approach tendencies toward food in contexts where food was task-irrelevant (i.e., as indexed by the AST). Following a positive mood induction, restrained eating was associated with stronger approach tendencies toward food, whereas in the sad mood condition, this effect was reversed (i.e., the higher the RS score the less the approach bias for food). For the SRC, results point in the same direction, but only for high caloric food. This is in line with several studies pointing to increased food intake during positive emotions for eating- or weight concerned people (e.g., emotional and restrained eaters; Yeomans and Coughlan, 2009; Bongers et al., 2013)

One testable explanation of why positive emotions may lead to food indulgence is that happiness produces more impulsive and non-reflective processing. Especially in restrained eaters this may lead to heightened approach behavior inconsistent with conscious intentions (Schwarz and Bless, 1991). In other words, especially in restrained eaters, a positive mood might activate food-approach associations.

Following a negative mood induction, restrained eating was negatively correlated with automatic approach tendencies toward food. This seems inconsistent with earlier studies on mood and eating showing that a negative mood leads to increased food intake in restrained eaters (e.g., Schotte et al., 1990). However, a distinction can be made between approach tendencies and actual eating. Possibly, both a positive and negative mood could lead to eating in restrained eaters, but eating in a negative mood might be more due to intentional processes (in order to improve mood), whereas eating in a positive mood might be more strongly under the influence of more automatic processes such as automatic approach tendencies.

The present findings suggest that people by default (not restrained) show a heightened approach tendency toward food when in a negative mood. Food might play a role in affect regulation. The affect regulation model posits that dysphoric individuals eat in an effort to provide comfort or distraction from negative emotions (Stice et al., 2005). If food is regularly consumed to end a negative feeling then eating will become associated with aspects of the preceding negative state (Deutsch and Strack, 2006). One explanation of why this sad mood did not lead tot stronger approach tendencies in high restrained eaters, might be that especially for this group the sad mood might have heightened the accessibility of negative associations with food-intake (e.g., weight gain) thereby promoting avoidance instead of approach of food.

### Approach Bias, Craving, and Food Intake

There was no effect of mood and restraint on actual food intake. This is inconsistent with prior research (Schotte et al., 1990), which showed that negative affect triggered overeating in restrained eaters. There are, however, several differences between the studies, including the mood induction procedure (watching a frightening film vs. listening to sad music) and the measure of food intake, which might explain the differences in outcomes.

There was a positive relationship between craving for high caloric food and both approach bias as measured with the SRC and the AST, thereby supporting the validity of the tasks. Although both tasks were significantly correlated with craving, there was no significant relationship between the SRC and the AST food approach indices. The finding is in line with the idea that each of these tasks measures approach bias in different types of contexts; and are also predictive for different real life situations. The finding that previous studies on approach bias in restrained eating showed apparently inconsistent results, might therefore (at least partly) be due to the fact that different paradigms were used: food as a task-irrelevant (e.g., Veenstra and de Jong, 2010) vs. food a task-relevant feature (e.g., Fishbach and Shah, 2006).

Approach bias for high and low caloric food was highly correlated within tasks, and there was no interaction effect between food type and mood on approach tendencies. This indicates that there is an effect of food in general and not of specifically high or low caloric food. The relationship between approach bias and actual eating during the taste task was only present (as a non-significant trend) in the AST. In the taste task the participant was instructed to complete questions about the taste and quality of the food. In this task eating the food itself was not task-relevant because someone might be less actively thinking about the question whether to choose/approach the food or not, and how much to eat. The food may nevertheless elicit automatic approach tendencies toward food thereby leading to more food intake. The AST, in which the food is task-irrelevant, might thus better mimic approach behavior during the current taste task than the SRC-task. The mood induction did not directly result in differences in food intake. Possibly, the mood manipulation or the taste task was not sufficiently sensitive, and future studies are needed to further explore the relationship between mood and actual food intake.

### Limitations and Future Research

In the current study, a design was chosen with a fixed order of the two computer tasks measuring approach bias. As described earlier, this was chosen because we anticipated less carry-over effects from a task with food as task-irrelevant feature to a task with food as a task-relevant feature than vice versa. Prolonged exposure to food items might possibly enhance the vulnerability for bottom up interference of food item tasks, especially in restrained eaters. Or, in contrast, this exposure could lead to habituation and consequently effects of the computer tasks will be less pronounced. Since the AST was administered first in all participant, the possible influence would only be present in the SRC. Because an approach bias effect was found in SRC, even for unrestrained eaters, the habitation hypothesis seems not very plausible. To study carry-over effects and influence of time, a design with a balanced instead of a fixed order of computer tasks could be used in future studies.

Although differences were found between the various mood states in interaction with individual differences in restraint status, an analysis of the effectiveness of the mood induction did not show that the neutral condition differed significantly from the other conditions in self-reported mood states. One explanation for this could be that the mood ratings were completed after the ASTs and did not immediately follow the mood induction procedure. The effects of the mood induction might have been high initially, but might have decreased over time, resulting in back to normal levels of self-reported mood states. The finding that automatic approach tendencies did vary as a function of mood condition supports this view. The most plausible explanation for the differential effects is a change of mood by the mood induction. There were several reason for choosing this design instead of a mood rating directly after the mood indication. Firstly, this mood procedure had been shown effective in various previous studies (e.g., Werthmann et al., 2014), so there was no reason to assume that this would be different in the current study. Secondly, reflecting on mood could possibly influence the mood state itself. We anticipated that with doing the AST immediately following the mood induction the effect of the mood induction would be maximized.

A limitation of the current study is that our sample was predominantly lean and young, so findings cannot be generalized to overweight/obese participants or to a wide age range. Secondly, we did not compare groups receiving course credits vs. money. Although there seem no strong reasons to assume that both types of reimbursement would result in different response patterns, we cannot rule this out. Furthermore, the current study lacked sufficient power to detect small effects. Therefore, it seems important to replicate the current findings in a larger sample with adequate power to also reliably detect relatively small effects of both the mood induction and participants’ restrained status. Lastly, the design of the study to answer the question whether approach bias was associated with craving and actual eating was suboptimal because the mood induction possibly caused extra variance.

## Conclusion

The current study showed that, in line with dietary goals, high-restrained eaters displayed less approach tendencies than low restrained eaters when food was task-relevant. However, when in a positive mood and when food was task-irrelevant, automatic approach tendencies were heightened, which may interfere with restrained eaters’ goal of restricting food-intake. Thus, specifically outside regular mealtimes positive mood might lower the threshold for overeating in restrained individuals.

## Ethics Statement

This study was approved by the Ethic committee of the psychology department of the University of Groningen. Informed consent was signed prior to the experiment.

## Author Contributions

RN, AR, BO, and PdJ made substantial contributions to the conception and design of the work; of the acquisition, analysis, and interpretation of data for the work; drafting the work or revising it critically for important intellectual content; final approval of the version to be published; and agreement to be accountable for all aspects of the work in ensuring that questions related to the accuracy or integrity of any part of the work are appropriately investigated and resolved.

## Conflict of Interest Statement

The authors declare that the research was conducted in the absence of any commercial or financial relationships that could be construed as a potential conflict of interest.
